# Supplementary material for: Impact of 2′-deoxyribo-purine substrates on nonenzymatic RNA template-directed primer extension
Source: Nucleic Acids Res. 2025 Nov 20;53(21):gkaf1228. doi: 10.1093/nar/gkaf1228 (PMC12630140; doi:10.1093/nar/gkaf1228)
Supplement: gkaf1228_Supplemental_File [file gkaf1228_supplemental_file.pdf]

Supplementary Data

Impact of 2′-deoxyribo-purine substrates on nonenzymatic RNA template-directed primer extension

Ziyuan Fang[1], Orhan Acikgoz[1], Xiwen Jia[1,2], Jahmyl Essex[1], Ruby Wen[1], Jack W. Szostak[1]*

[1] Howard Hughes Medical Institute, Department of Chemistry, The University of Chicago, Chicago, IL 60637, USA
[2] Department of Chemistry and Chemical Biology, Harvard University, 12 Oxford Street, Cambridge, MA 02138, USA

* To whom correspondence should be addressed. Email: jwszostak@uchicago.edu

Present Address:
Ziyuan Fang, Quantum-Si, Branford, CT, 06405, USA.
Xiwen Jia, Novo Nordisk, Lexington, MA, 02421, USA.

1. Synthesis and characterization of homo-bridged-dinucleotides

The 5′-5′-2-aminoimidazolium-bridged-dinucleotides were synthesized following a previously reported procedure (1,2). Among them, A*A, C*C, G*G, U*U, I*I, s^2C*s^2C, s^2U*s^2U have been described in prior publications (2), whereas dI*dI, dA*dA and dG*dG were synthesized for the first time in this study. Details of the nuclear magnetic resonance (NMR) spectra and high-resolution mass spectrometry (HRMS) of dI*dI, dA*dA and dG*dG are provided below.

1.1 1,3-di-(3′-deoxyinosine-5′phosphoryl)-2-aminoimidazolium (dI*dI)

^{1}H NMR (600 MHz, D_2O) δ 8.19 (s, 2H), 8.15 (s, 2H), 6.65 (s, 2H), 6.34 (t, *J* = 6.5 Hz, 2H), 4.64 (m, 2H), 4.13-4.05 (m, 4H), 4.05-4.00 (m, 2H), 2.79 (m, 2H), 2.54 (m, 2H). Peaks corresponding to residual TEAB observed at 3.21 and 1.29 ppm.

^{31}P NMR (162 MHz, D_2O) δ -12.91. (The purity is 100% with no detectable activated monomer peak)

HRMS (Q-TOF) m/z: $[M - H]^-$ Calcd. for $C_{23}H_{26}N_{11}O_{12}P_2$ 710.1238; Found: 710.1307.

1.2 1,3-di-(3′-deoxyadenosine-5′phosphoryl)-2-aminoimidazolium (dA*dA)

^{1}H NMR (600 MHz, D_2O) δ 8.12 (s, 2H), 8.10 (s, 2H), 6.65 (s, 2H), 6.29 (t, *J* = 6.5 Hz, 2H), 4.59 (m, 2H), 4.12-4.06 (m, 4H), 4.01-3.96 (m, 2H), 2.70 (m, 2H), 2.53 (m, 2H). Peaks corresponding to residual TEAB observed at 3.21 and 1.29 ppm.

^{31}P NMR (162 MHz, D_2O) δ -12.80. (The purity is 100% with no detectable activated monomer peak)

HRMS (Q-TOF) m/z: $[M - H]^-$ Calcd. for $C_{23}H_{28}N_{13}O_{10}P_2$ 708.1558; Found: 708.1685.

1.3 1,3-di-(3′-deoxyguanosine-5′phosphoryl)-2-aminoimidazolium (dG*dG)

^{1}H NMR (600 MHz, D_2O) δ 7.86 (s, 2H), 6.62 (s, 2H), 6.16 (t, *J* = 6.7 Hz, 2H), 4.61 (m, 2H), 4.1-4.05 (m, 4H), 3.98 (m, 2H), 2.74 (m, 2H), 2.47 (m, 2H). Peaks corresponding to residual TEAB observed at 3.21 and 1.29 ppm.

^{31}P NMR (162 MHz, D_2O) δ -12.80. (The purity is 100% with no detectable activated monomer peak)

HRMS (Q-TOF) m/z: $[M - H]^-$ Calcd. for $C_{23}H_{28}N_{13}O_{12}P_2$ 740.1456; Found: 740.1551.

2. Supplementary Figures and Tables

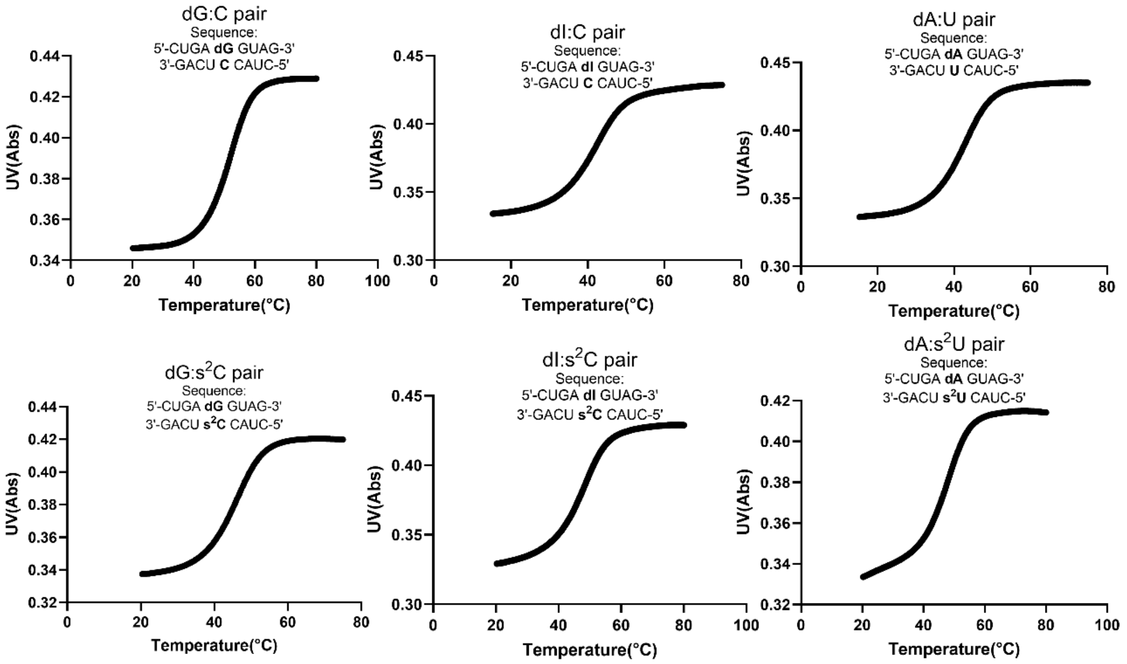

Figure S1. Representative melting curves collected during the thermal denaturation of 5 μM oligonucleotide in 10 mM Tris-HCl 8.0, 1 M NaCl, and 2.5 mM EDTA.

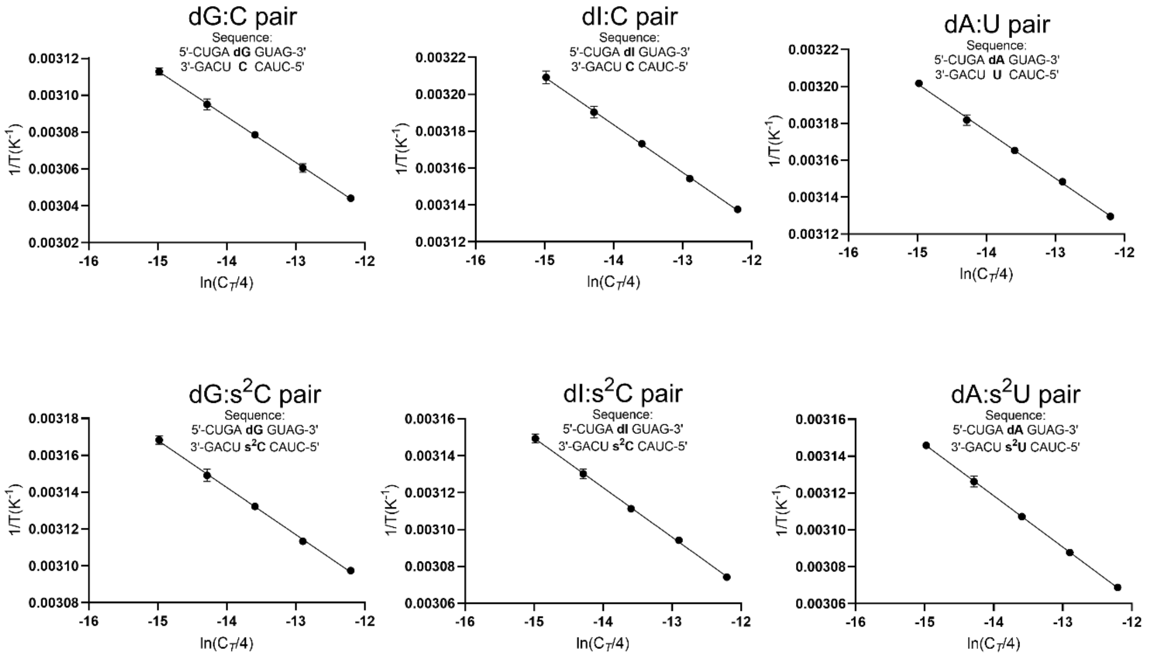

Figure S2. Linear least-squared fits of a Van't Hoff plot of inverse melting temperature (T_m^{-1}) collected from optical melts at different oligonucleotide concentrations.

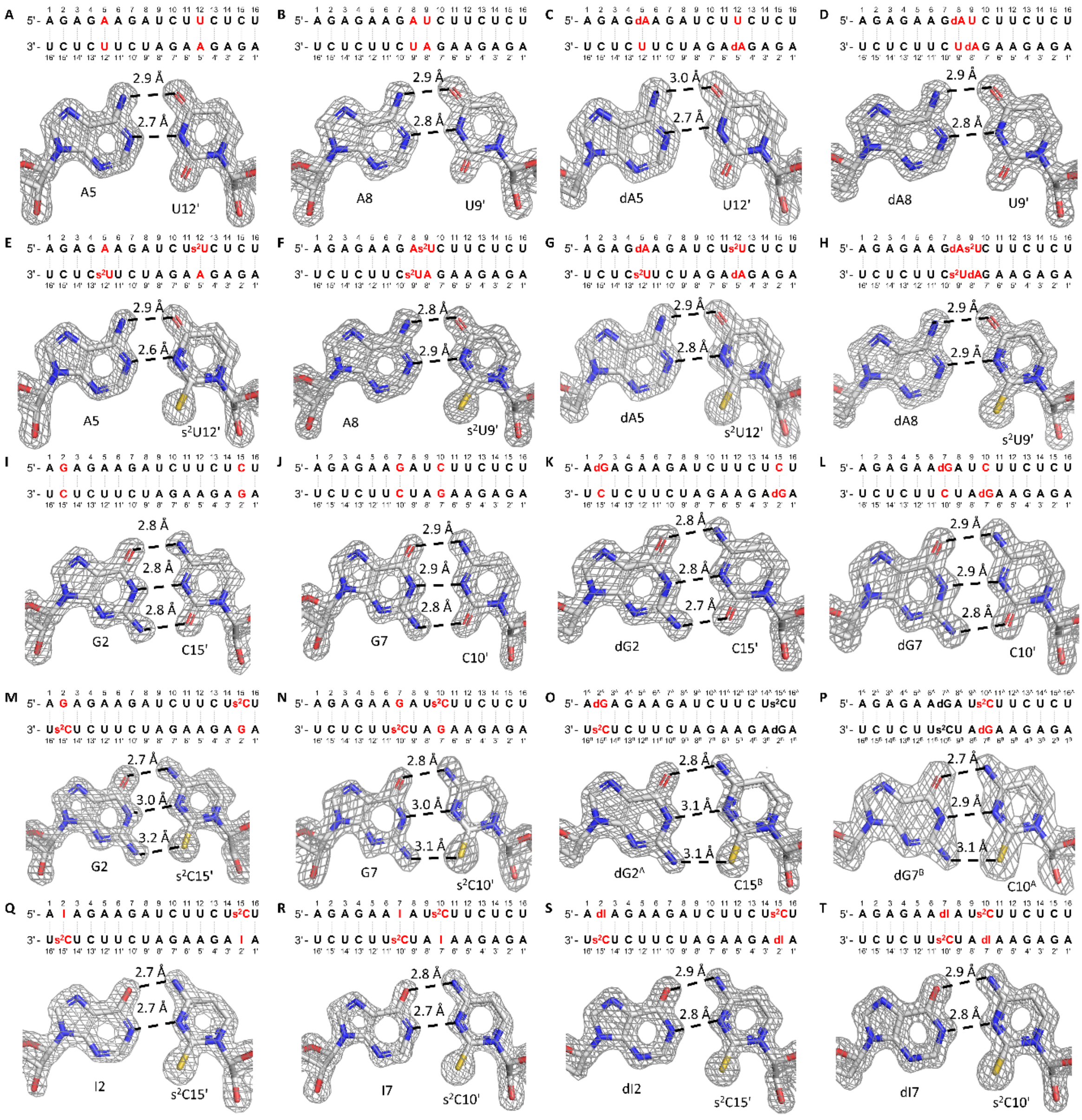

Figure S3. Ribo- and deoxy-purines involved base pairs in 16mer RNA duplex crystal structures. Sequence context and density maps are shown for the following base pairs: (A) A5:U12 in Native16; (B) A8:U9 in Native16; (C) dA5:U12 in dAU1; (D) dA8:U9 in in dAU2; (E) A5:s^{2}U12 in AUS1; (F) A8:s^{2}U9 in AUS2; (G) dA5:s^{2}U12 in dAUS1; (H) dA8:s^{2}U9 in dAUS2; (I) G2:C15 in Native16; (J) G7:C10 in Native16; (K) dG2:C15 in dGC1; (L) dG7:C10 in dGC2; (M) G2:s^{2}C15 in GCS1; (N) G7:s^{2}C10 in GCS2; (O) dG2^A:s^{2}C15^B in dGCS1; (P) dG7^B:s^{2}C10^A in dGCS2; (Q) I2:s^{2}C15 in ICS1; (R) I7:s^{2}C10 in ICS2; (S) dI2:s^{2}C15 in dICS1; (T) dI7:s^{2}C10 in dICS2. Gray mesh represents the corresponding 2F$_o$-F$_c$ omit maps for water molecules contoured at 1.5 σ. The structures of Native16, AUS1, AUS2, GCS1, GCS2, ICS1, and ICS2 were previously reported in reference (2), and the figures presented here are derived from the corresponding PDB entries: 9CSO, 9CSP, 9CSQ, 9CSR, 9MDW, 9MDX, and 9MDY, respectively.

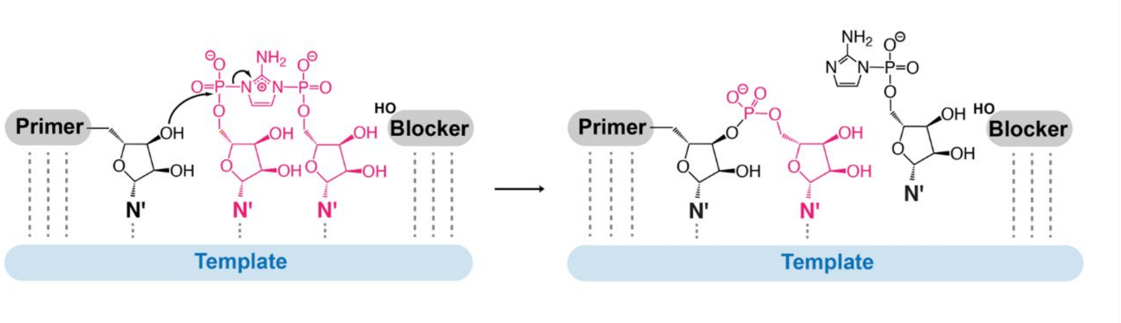

Figure S4. Mechanism of bridged dinucleotide (N*N) primer extension within the template-primer-blocker complex

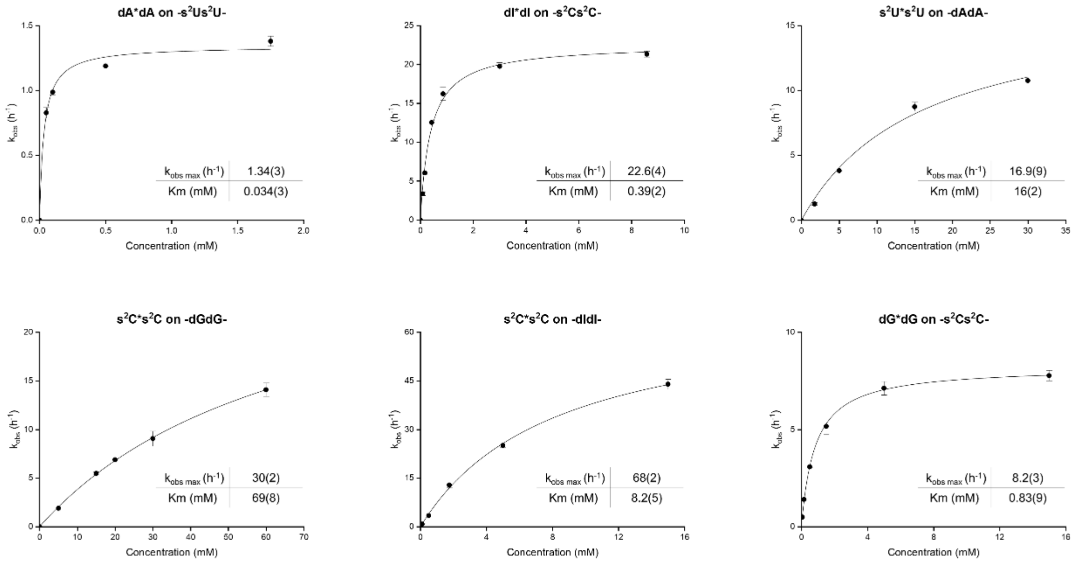

Figure S5. Michaelis-Menten curves for primer extension reactions with bridged substrates on the indicated template sequences.

Table S1. Oligo Sequences for Crystallization

Oligo	Sequence
Native16	5′-AGA GAA GAU CUU CUC U-3′
dAU1	5′-AGA G**dA**A GAU CU**U** CUC U-3′
dAU2	5′-AGA GAA G**dAU** CUU CUC U-3′
AUS1	5′-AGA G**A**A GAU CU**s^{2}U** CUC U-3′
AUS2	5′-AGA GAA G**As^{2}U** CUU CUC U-3′
dAUS1	5′-AGA G**dA**A GAU CU**s^{2}U** CUC U-3′
dAUS2	5′-AGA GAA G**dAs^{2}U** CUU CUC U-3′
dGC1	5′-A**dG**A GAA GAU CUU CU**C** U-3′
dGC2	5′-AGA GAA **dG**AU **C**UU CUC U-3′
GCS1	5′-A**G**A GAA GAU CUU CU**s^{2}C** U-3′
GCS2	5′-AGA GAA **G**AU **s^{2}C**UU CUC U-3′
dGCS1	5′-A**dG**A GAA GAU CUU CU**s^{2}C** U-3′
dGCS2	5′-AGA GAA **dG**AU **s^{2}C**UU CUC U-3′
ICS1	5′-A**I**A GAA GAU CUU CU**s^{2}C** U-3′
ICS2	5′-AGA GAA **I**AU **s^{2}C**UU CUC U-3′
dICS1	5′-A**dI**A GAA GAU CUU CU**s^{2}C** U-3′
dICS2	5′-AGA GAA **dI**AU **s^{2}C**UU CUC U-3′
14mer	5′-$_{m}C_{m}C_{m}C$ GAC UUA AGU CG-3′

Table S2. Optimized Conditions for Crystallization

Oligo	Optimized crystallization conditions
dAU1	0.08 M Sodium chloride, 0.012 M Potassium chloride, 0.02 M Magnesium chloride hexahydrate, 0.04 M Sodium cacodylate trihydrate pH 6.0, 30% v/v (+/-)-2-Methyl-2,4-pentanediol, 0.012 M Spermine tetrahydrochloride.
dAU2	0.08 M Sodium chloride, 0.02 M Magnesium chloride hexahydrate, 0.04 M Sodium cacodylate trihydrate pH 5.5, 35% v/v (+/-)-2-Methyl-2,4-pentanediol, 0.002 M Hexammine cobalt(III) chloride.
dAUS1	0.2 M Magnesium chloride hexahydrate, 0.1 M HEPES sodium pH 7.5, 30% v/v Polyethylene glycol 400.
dAUS2	0.02 M Calcium chloride dihydrate, 0.1 M Sodium acetate trihydrate pH 4.6, 30% v/v (+/-)-2-Methyl-2,4-pentanediol.
dGC1	25 % v/v Polyethylene glycol monomethyl ether 550, 50 mM HEPES pH 7.0, 10 mM Magnesium chloride
dGC2	0.4 M Sodium chloride, 0.12 M Calcium chloride, 20 mM MES pH 5.8, 27% v/v (+/-)-2-Methyl-2,4-pentanediol.
dGCS1	0.1 M BIS-TRIS pH 5.5, 2.0 M Ammonium sulfate.
dGCS2	0.1 M MES pH 6.5, 0.06 M Manganese (II) chloride, 15.00 % w/v Polyethylene glycol 20,000
dICS1	0.2 M Magnesium chloride hexahydrate, 0.1 M HEPES sodium pH 7.5, 30% v/v Polyethylene glycol 400.
dICS2	0.2 M Calcium chloride dihydrate, 0.05 M HEPES sodium pH 7.5, 28% v/v Polyethylene glycol 400, 0.002 M Spermine
RNAsub	0.05 M HEPES pH 7.0, 0.2 M Ammonium acetate, 0.15 M Magnesium acetate, 10% w/v Polyethylene glycol 6,000.
DNAsub	0.05 M HEPES pH 7.0, 0.2 M Ammonium acetate, 0.15 M Magnesium acetate, 10% w/v Polyethylene glycol 6,000.

Table S3. Data Collection Statistics

Sequences	dAU1	dAU2	dAUS1	dAUS2
PDB code	9OKS	9OKT	9OKU	9OKV
Beamline	ALS 5.0.1	ALS 5.0.1	ALS 5.0.1	ALS 5.0.1
Wavelength (Å)	0.97741	0.97741	0.97741	0.97741
Space group	H32	H32	H32	H32
Unit cell parameters (Å, °)	41.2, 41.2, 125.6, 90, 90, 120	41.0, 41.0, 123.4, 90, 90, 120	41.2, 41.2, 124.6, 90, 90, 120	41.1, 41.1, 124.3, 90, 90, 120
Resolution range (Å)	50.0-1.50 (1.53-1.50)	50.0-1.32 (1.34-1.32)	50.0-1.48 (1.51-1.48)	50.0-1.23 (1.25-1.23)
Unique reflections	6909 (334)	9461 (417)	7122 (344)	11714 (388)
Completeness (%)	100 (100)	96.9 (88.7)	100 (100)	96.2 (62.3)
R_{merge} (%)	6.7 (51.0)	6.0 (51.1)	6.1 (57.3)	4.0 (21.4)
$\langle I/\sigma(I)\rangle$	30.0 (4.0)	30.3 (4.0)	33.0 (3.5)	52.0 (5.2)
Redundancy	8.7 (8.2)	9.4 (8.6)	9.1 (8.6)	8.3 (3.4)

Sequences	dGC1	dGC2	dGCS1	dGCS2
PDB code	9OKW	9OKX	9OKY	9OKZ
Beamline	ALS 5.0.3	ALS 5.0.3	ALS 5.0.3	ALS 8.2.1
Wavelength (Å)	0.97648	0.97648	0.97648	1.00003
Space group	H32	H32	H32	P31
Unit cell parameters (Å, °)	41.3, 41.3, 124.6, 90, 90, 120	41.5, 41.5, 124.6, 90, 90, 120	43.4, 43.4, 256.4, 90, 90, 120	42.5, 42.5, 122.7, 90, 90, 120
Resolution range (Å)	50.0-1.54 (1.57-1.54)	50.0-1.38 (1.40-1.38)	50.0-1.48 (1.51-1.48)	50.0-1.92 (1.95-1.92)
Unique reflections	6327 (294)	8922 (405)	15145 (756)	18877 (919)
Completeness (%)	99.9 (100)	99.5 (96.2)	93.2 (93.6)	99.3 (99.4)
R_{merge} (%)	8.7 (33.5)	6.7 (49.4)	7.0 (50.6)	4.4 (57.0)
$\langle I/\sigma(I)\rangle$	24.5 (3.8)	26.0 (2.8)	20.2 (2.7)	32.8 (2.8)
Redundancy	8.5 (4.9)	7.2 (5.2)	8.2 (5.0)	5.0 (4.9)

Sequences	dICS1	dICS2	RNAsub	DNAsub
PDB code	9OL0	9OL1	9OL2	9OL3
Beamline	NSLS-II 17-ID-2	NSLS-II 17-ID-2	APS 23-ID-B	APS 23-ID-B
Wavelength (Å)	0.97933	0.97933	1.033175	1.033175
Space group	H32	H32	P321	P321
Unit cell parameters (Å, °)	41.3, 41.3, 124.4, 90, 90, 120	40.9, 40.9, 125.0, 90, 90, 120	49.6, 49.6, 81.9, 90, 90, 120	48.2, 48.2, 82.7, 90, 90, 120
Resolution range (Å)	34.4-1.15 (1.17-1.15)	34.1-1.24 (1.26-1.24)	50-1.53 (1.56-1.53)	50-1.56 (1.59-1.56)
Unique reflections	14617 (819)	11727 (572)	17783 (757)	15265 (433)
Completeness (%)	98.1 (94.5)	100 (100)	96.9 (84.9)	92.4 (55.2)
R_{merge} (%)	6.1 (126)	12.8 (200)	7.9 (41.5)	5.2 (43.8)
$\langle I/\sigma(I) \rangle$	14.9 (1.5)	12.5 (2.0)	15.9 (2.8)	49.6 (2.6)
Redundancy	9.8 (8.2)	17.6 (14.7)	5.9 (4.8)	6.5 (3.7)

Table S4. Data Refinement Statistics

Sequences	dAU1	dAU2	dAUS1	dAUS2
PDB code	9OKS	9OKT	9OKU	9OKV
RNA strands per asymmetric unit	1	1	1	1
Resolution range (Å)	41.86-1.50	34.13-1.32	41.52-1.48	41.46-1.23
Number of reflections	6402	8918	6761	11009
R_{work} (%)	16.6	18.3	17.0	18.3
R_{free} (%)	21.0	20.6	21.1	22.7
Bond length R.M.S. (Å)	0.010	0.010	0.011	0.013
Bond angle R.M.S. (°)	2.01	2.30	1.98	2.04
Average B-factors (Å^2)	10.3	11.9	11.5	12.1

Sequences	dGC1	dGC2	dGCS1	dGCS2
PDB code	9OKW	9OKX	9OKY	9OKZ
RNA strands per asymmetric unit	1	1	2	6
Resolution range (Å)	41.53-1.54	41.54-1.38	30.33-1.48	36.81-1.92
Number of reflections		7991	14221	16740
R_{work} (%)	19.9	17.7	22.0	18.7
R_{free} (%)	24.8	21.5	24.8	25.7
Bond length R.M.S. (Å)	0.002	0.008	0.012	0.007
Bond angle R.M.S. (°)	0.48	1.86	2.21	2.06
Average B-factors (Å^2)	14.8	10.6	12.2	14.9

Sequences	dICS1	dICS2	RNAsub	DNAsub
PDB code	9OL0	9OL1	9OL2	9OL3
RNA strands per asymmetric unit	1	1	2	2
Resolution range (Å)	34.37-1.15	34.08-1.24	42.95-1.53	41.78-1.56
Number of reflections	14594	10551	16762	14497
R_{work} (%)	20.3	18.6	19.8	20.4
R_{free} (%)	23.3	22.9	23.4	24.9
Bond length R.M.S. (Å)	0.013	0.012	0.020	0.020
Bond angle R.M.S. (°)	1.77	2.08	3.04	2.93
Average B-factors (Å^2)	20.0	12.6	15.9	48.1

Table S5. Combination of the primer, template, blocker and complementary oligonucleotides used in the Michaelis-Menten analysis of primer extension reactions.

Bridged dinucleotide	Template Sequence	Primer	Template	Blocker	Complementary DNA
s^2U*s^2U	-AA-	DL-30	LA-121	LA-111	dCLA-121
s^2U*s^2U	-dAdA-	DL-30	dAT26	LA-111	dCLA-121
s^2C*s^2C	-GG-	DL-30	LA-124	LA-111	dCLA-124
s^2C*s^2C	-dGdG-	DL-30	dGT26	LA-111	dCLA-124
s^2C*s^2C	-II-	DL-30	IIT26	LA-111	dCLA-123
s^2C*s^2C	-dIdI-	DL-30	dIT26	LA-111	dCLA-123
A*A	-s^2Us^2U-	DL-30	S2UT	LA-111	dCLA-122
dA*dA	-s^2Us^2U-	DL-30	S2UT	LA-111	dCLA-122
G*G	-s^2Cs^2C-	DL-30	S2CT	LA-111	dCLA-123
dG*dG	-s^2Cs^2C-	DL-30	S2CT	LA-111	dCLA-123
I*I	-s^2Cs^2C-	DL-30	S2CT	LA-111	dCLA-123
dI*dI	-s^2Cs^2C-	DL-30	S2CT	LA-111	dCLA-123

Table S6. Sequences of oligonucleotides used in the Michaelis-Menten analysis of primer extension reactions.

Name	Role	Source	Type	Sequence (5′→ 3′)
DL-30	Primer	IDT	RNA	/FAM/AGU GAG UAA CGG
LA-111	Blocker	IDT	RNA	G AUG UCA GAU AU
IIT26	Template	In-house	RNA	AU AUC UGA CAU C**II** CCG UUA CUC ACU
dIT26	Template	In-house	RNA	AU AUC UGA CAU C**dIdI** CCG UUA CUC ACU
LA-121	Template	IDT	RNA	AU AUC UGA CAU C**AA** CCG UUA CUC ACU
dAT26	Template	In-house	RNA	AU AUC UGA CAU C**dAdA** CCG UUA CUC ACU
dCLA-121	Complementary strand	IDT	DNA	AGT GAG TAA CGG **TT**G ATG TCA GAT AT
LA-122	Template	IDT	RNA	AU AUC UGA CAU C**UU** CCG UUA CUC ACU
dCLA-122	Complementary strand	IDT	DNA	AGT GAG TAA CGG **AA**G ATG TCA GAT AT
LA-123	Template	IDT	RNA	AU AUC UGA CAU C**CC** CCG UUA CUC ACU
dCLA-123	Complementary strand	IDT	DNA	AGT GAG TAA CGG **GG**G ATG TCA GAT AT
LA-124	Template	IDT	RNA	AU AUC UGA CAU C**GG** CCG UUA CUC ACU
dGT26	Template	In-house	RNA	AU AUC UGA CAU C**dGdG** CCG UUA CUC ACU
dCLA-124	Complementary strand	IDT	DNA	AGT GAG TAA CGG **CC**G ATG TCA GAT AT
2SCT	Template	In-house	RNA	AU AUC UGA CAU C**s^{2}Cs^{2}C** CCG UUA CUC ACU
2SUT	Template	In-house	RNA	AU AUC UGA CAU C**s^{2}Us^{2}U** CCG UUA CUC ACU

Reference

1. Ding, D., Zhou, L., Giurgiu, C. and Szostak, J.W. (2022) Kinetic explanations for the sequence biases observed in the nonenzymatic copying of RNA templates. *Nucleic Acids Research*, **50**, 35-45.
2. Fang, Z., Jia, X., Xing, Y. and Szostak, J.W. (2025) Nonenzymatic RNA copying with a potentially primordial genetic alphabet. *Proceedings of the National Academy of Sciences*, **122**, e2505720122.
